# Hope on the Horizon? Aptamers in Diagnosis of Invasive Fungal Infections

**DOI:** 10.3390/genes15060733

**Published:** 2024-06-03

**Authors:** Sadegh Khodavaisy, Jianping Xu

**Affiliations:** 1 Department of Biology, McMaster University, Hamilton, ON L8S 4K1, Canada; khodavas@mcmaster.ca; 2 Department of Medical Parasitology and Mycology, School of Public Health, Tehran University of Medical Sciences, Tehran 1417613151, Iran

**Keywords:** aptamers, invasive fungal infections, diagnosis, treatment

## Abstract

Despite remarkable advances in the diagnosis of invasive fungal infections (IFIs), rapid, specific, sensitive, and cost-effective detection methods remain elusive. Due to their stability, ease of production, and specificity to signature molecules of fungal pathogens, short single-stranded sequences of DNA, RNA, and XNA, collectively called aptamers, have emerged as promising diagnostic markers. In this perspective, we summarize recent progress in aptamer-based diagnostic tools for IFIs and discuss how these tools could potentially meet the needs and provide economical and simple solutions for point-of-care for better management of IFIs.

## 1. Introduction

Invasive Fungal Infections (IFIs) have become a significant global healthcare concern among immunocompromised individuals, including persons with hematological malignancies, solid organ transplant recipients, and those treated with broad-spectrum antibiotics and corticosteroids [1]. Vulnerability to IFIs has also been reported among patients admitted to intensive care units (ICUs) with invasive procedures and indwelling catheter, AIDS patients, diabetics, burn patients, and severe COVID-19 patients [2]. Patients with IFIs have high mortality rates. Several factors contribute to such high rates, including difficulties associated with diagnosis of IFIs, host immune deficiency, limited treatment options, and the development of drug resistance [3]. Early and accurate diagnosis of IFIs is crucial for efficient management. However, current diagnosis of IFIs with conventional methods is a lengthy process, and suffers from one or more of the technical limitations [4]. An ideal technology should be rapid, sensitive, specific, and cost effective [5].

Over the last twenty years, a diversity of molecular techniques has been developed for diagnosing fungal pathogens. Most of these are based on polymerase chain reaction (PCR). PCR-based methods are typically very sensitive and rapid, but the principal shortcomings in applying these assays to the clinical setting include expensive reagents and instruments, dedicated space, experienced personnel, and false-positive results from background DNA contamination [6]. In general, PCR diagnostics is a highly complex technique that are not often effective in differentiating among active infections, and colonisations or asymptomatic carriers. On the other hand, the other major category of diagnostic methods based on serology requires the availability of specific antibodies with long and expensive production process and they often suffer cross-reactivity (low specificity) and limited sensitivity [7]. Therefore, the development of fast, cost-effective, reproducible, and reliable novel methods remains a necessity. 

Recently, aptamer-based diagnostic markers have attracted increasing attention [8]. Aptamers are short single-stranded sequences of DNA, RNA, xeno nucleic acids (XNA, nucleic acids with a backbone not made of ribose or deoxyribose), or even peptide that can bind one of a family of specific target molecules. Aptamers can be a good alternative to antibodies in target recognition due to their advantages in small size, flexible structure, greater specificity, easier chemical modifications, low immunogenicity, and greater stability [9]. This is especially true for DNA aptamers where ease of production, affordable price, flexible design, and thermal and chemical stability are well known [10]. In addition, DNA (and other nucleic acid) aptamers can be modified by fluorescent dyes for visual detection or used in combination with various kinds of biosensing platforms, including fluorometry, colorimetry, chemiluminometry, and electrochemistry [11,12]. Together, the feasibility of integration into various diagnostic platforms makes aptamers ideal markers for developing simple, rapid, and cost-effective laboratory diagnostic platforms, such as biosensors, microarrays, and lateral flow assays. Indeed, over the past decades, nucleic acid aptamers have been used in a wide range of diagnostic applications. In this minireview, we first briefly describe how aptamers are commonly developed. This is then followed by a summary of recent progress in the development of aptamers for diagnosing invasive fungal infections.

## 2. Aptamer and SELEX 

**Figure 1:**
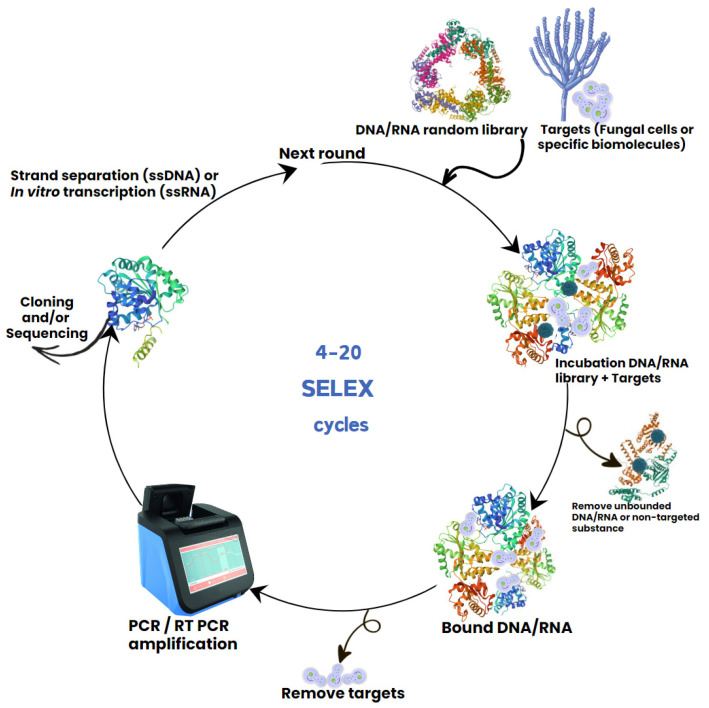
Schematic presentation of DNA aptamer selection using SELEX method for diagnosis and treatment of fungal infections.

Nucleic acid aptamers are typically single-stranded deoxyribonucleic acid (ssDNA) or ribonucleic acid (RNA) molecules that can form relatively stable secondary and/or tertiary structures and bind to a variety of specific targets [13]. High affinity aptamers typically contain 25–100 nucleotides that can wrap around a small molecule target or gaps within the surface of whole organisms, such as pathogenic bacteria or fungal cells. Aptamers are commonly selected through an in vitro procedure via the systematic evolution of ligands through exponential enrichment (SELEX), which was first demonstrated in 1990 [14,15]. SELEX uses reiterative screening of a random oligonucleotide library to identify high-affinity binders to a chosen target, which may be a peptide or other molecules on the surface of cells [16]. Aptamers are screened by a repeated cycle that includes three steps: binding of an oligonucleotides library to target molecules or whole cells; removal of unbound oligonucleotides; and amplification of the bound oligonucleotides by PCR (Figure 1) [17]. This ensures that only sequences with high affinity to target molecule are enriched and selected for subsequent cycles. After the library enrichment, the nucleotide sequences of selected oligonucleotides are further analysed. SELEX has been applied to develop many aptamers with specificity to different pathogenic microbes, from viral to bacterial and fungal pathogens. These studies also revealed that low diversity of oligonucleotide library sequences, non-specific binding, reagents contamination, the type and frequency of target molecules in the sample, and changes in cellular states on target cells can all negatively affect the enrichment of the aptamer sequences [18].

## 3. Aptamers’ Applications in Fungal Infections

Compared to the relatively large number of aptamers developed for detecting pathogenic bacteria and viruses [17], relatively few aptamers have been reported for fungal pathogens. Table 1 summarizes the characteristics of aptamers that have been developed for diagnostics or therapeutics for fungal infections. 

A major target of aptamers for fungal pathogens is (1→3) β-D-glucan (BDG), a polysaccharide and a predominant and specific constituent of the cell wall in most fungi. BDG can be found in serum and other sterile samples during invasive infections by a variety of fungi, such as *Aspergillus fumigatus* and *Candida albicans,* and serve as a biomarker for diseases like invasive aspergillosis and invasive candidiasis. Tang et al. developed two high-affinity DNA aptamers (AD1 and AU1) to specifically recognize BDG in *C. albicans* with a low degree of polymerization [8]. They detected BDG in serum samples by using a double-aptamer sandwich enzyme-linked oligonucleotide assay (ELONA) with high sensitivity (92.31%) and high specificity (91.94%) for the diagnosis of invasive candidiasis or candidemia, showcasing their potential as valuable tools for early and accurate detection of fungal pathogens. Similarly, Hua et al. also developed a visual diagnostic method with a novel aptamer-G-quadruplex/hemin self-assembling color system (AGSCS) (G-quadruplex DNAzyme for a catalytic unit) for rapid, accurate, and convenient diagnosis of IFIs [19]. This diagnostic method showed a linear detection range for BDG between 1.6 pg/mL and 400 pg/mL on a microplate reader with high sensitivity (92.68%) and specificity (89.65%), higher than that of commercial glucan test. Furthermore, Clack et al. created a rapid and user-friendly method with aptamer-tagged gold-core-shell nanoparticles for the early detection of *C. albicans* based on the presence of BDG [20]. This platform could detect *C. albicans* from individual yeast colonies without prior sample processing, dilution, or purification. Instead, in the presence of fungal cells (and thus BDG), nanoparticles aggregate, causing a redshift in the UV-visible absorbance, turning the color from pink/purple to blue. This technology has the potential to revolutionize the diagnosis of yeast infections and would be especially advantageous in remote and/or underdeveloped areas.

The above-mentioned aptamers targeting BDG exhibit both similarities and differences in their characteristics and applications [8,19,20]. They differ in their nucleotide sequences, binding affinities, and diagnostic applications. Indeed, species-specific aptamers are needed to accurately diagnose fungal pathogens. While aptamers offer promising alternatives to traditional diagnostic methods and potential for targeted treatments, the diverse range of aptamers targeting essentially the same molecule from different pathogens requires additional optimization for commercial development, including considerations for species-specificity and diagnostic accuracy. However, this diversity also underscores the importance of rigorous validation and continued efforts in aptamer development to fully realize their potential in the diagnosis and treatment of IFIs.

A polyclonal aptamer library allowed the identification of *Candida*-species, including of *C. albicans*, *C. auris* and *C. parapsilosis,* in monoculture or mixed culture. In this study, the aptamer library was functional when combined with several standard diagnostic techniques, such as flow-cytometry, fluorometric microtiter plate assay, and fluorescence microscopy [21]. Similarly, Milnes et al. described 11 selected aptamers targeting whole cells of *A. fumigatus* and *C. albicans,* without knowing their individual target molecules [22]. Seo et al. isolated several aptamers capable of binding to spores of three representative toxic/pathogenic *Aspergillus* species *A. fumigatus*, *A. flavus*, and *A. niger,* using cell-SELEX. They showed that aptamer Asp-3 with a high binding affinity for spores was likely to be targeting a cell surface protein, which could be an effective biorecognition element for the spores of these *Aspergillus* species [23]. Together, while the specific target molecules are not known, these studies showed the great potential of aptamers as diagnostic markers for human fungal pathogens.

However, due to large differences in antifungal susceptibilities among many species, species-specific aptamers are needed to provide accurate diagnosis and treatment option(s) for patients. For example, among species within genus *Candida*, some, such as *C. auris* and *C. tropicalism* have intrinsically high MICs to several commonly used antifungal drugs [24,25,26]. Indeed, high percentages of strains of *C. auris* are resistant to each of the four major categories of antifungal drugs (azoles, echinocandins, polyene, and flucytosine), with a significant proportion being resistant to all four major categories of drugs [24]. Large differences in their antifungal MICs among species in the genus *Aspergillus* have also been observed [25,26]. Aside from identifying the initial infecting agents, species-specific aptamers can also help with monitoring the effectiveness of antifungal treatments by measuring pathogen load in patients. Thus, due to their stability and ease of use, aptamer-based diagnostic systems are particularly valuable options in remote and resource-limited areas.

Structurally, the fungal cell wall is a dynamic composite of polysaccharides and glycoproteins that could provide fungi with biological roles and other biotechnological assets. In addition to BDG, other species-specific cell surface molecules, such as Galactomannan (GM) in *Aspergillus* spp., glucuronoxylomannan polysaccharide (GXM) in *Cryptococcus* spp., and surface colonization factor (SCF1) in *C. auris,* could be potential targets for aptamer binding [27,28]. These species-specific targets not only aid in accurate diagnosis but also hold promise for monitoring treatment efficacy using aptamers. Indeed, the identification and development of aptamers targeting species-specific cell surface molecules represent a promising avenue for enhancing the diagnosis and management of IFIs.

While different from the typical aptamers, RNA interference, also based on short single-stranded RNA, has been used to downregulate gene expression in a variety of organisms, including in fungal pathogens. Interestingly, targeted treatments using aptamers have been shown to increase the survival rate of patients suffering invasive microbial infections. Specifically, with high-affinity binding to cell surface molecules of microbial pathogens, aptamers can hinder microbial binding to host cells and potentially prevent pathogen infection of the host. This property is being exploited to help in delivering antimicrobial drugs directly to the site of infection, facilitating drug entry into pathogen cells, thus improving the effectiveness of treatments [18]. Aptamers can also help modulate the immune system to enhance host defenses, especially in immunocompromised patients [18]. Through targeted drug delivery, improved medication efficacy, enhanced immunity, and combinations of the above, aptamers have the potential to revolutionize the treatment of microbial infections, like IFIs. Indeed, aptamers have been used as therapeutic tools to combat chronic fungal infections, anti-biofilm formation, or conjugated with nanoparticles [18,29]. With the increasing prevalence of multidrug-resistant pathogens to conventional antimicrobial agents, aptamers represent a novel approach for managing infections caused by drug-resistant fungal pathogens. A recent study by Wiedman et al. highlighted the development of an azole drug-capturing oligonucleotide and graphene field effect transistor (GFET) device as a functional biosensor for drug monitoring of azole antifungal drugs in patients with IFIs [30]. 

**Table 1:** Summary of aptamers developed so far for the diagnostics and/or treatment of fungal infections.

Targets	Target Molecules	Aptamer Type/Name	Sequence of the Aptamer (5′ to 3′)	Secondary Structures of the Aptamer	Testing Platform	Specificity (%)	Sensitivity (%)	Reference
*Candida albicans*	(1→3)-β-D-glucans	dsDNA/AU1 and AD1	GCGGAATTCGAACAGTCCGAGCC-N60-GGGTCAATGCGTCATA	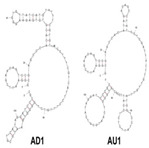	ELONA	91.94	92.31	Tang et al. [8]
*Candida albicans*	(1→3)-β-D-glucans	dsDNA/A1, A4, A5, and A6	TCTAGAATCCCAATCCCAATCCCA-50N-ACCCTAAAGCTT	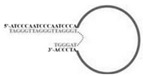	AGSCS	89.65	92.68	Hua et al. [19]
*Candida albicans*	(1→3)-β-D-glucans	dsDNA/AD1	GCGGAATTCGAACAGTCCGAGCCCACACGTGTGAGAAGGGTGTTATCATGTATTTCGTGTTCCTTTCGTCATTCCTTTGTCTGGGGTCAATGCGTCATAGGATCCCGCAAAAAAAAAA	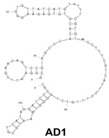	Aptamer binding to gold nanoparticles	-	-	Clack et al. [20]
*A. fumigatus* and *C. albicans*	Whole cell	DNA	Multiple aptamers	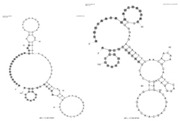	Fluorescence microscopy	-	-	Milnes [22]
*A. fumigatus*, *A. flavus*, and *A. niger*	Spores	ssDNA/Asp-3	CGTTTGGGCGGTATGAGTTCGGGGGTATACCGCAG	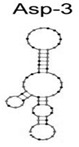	Cell-SELEX	-	-	Woo Seo et al. [23]
*Candida* species	Various	ssDNA	Polyclonal SELEX aptamer library	-	Flow-cytometry, Fluorometric microtiter plate assays, Fluorescence microscopy	-	-	Kneißle et al. [21]
Azole class antifungal drugs	Posaconazole	DNA/Rd 13, Rd 9	CGGGGGAGGCGGAGGGAGGGAGGACTGGGGCTTCATTGACGTTCTTCACAGTAGGGGTAAGGGCTTAGGTGGTTGGTGCCTG CGCGGGGAGGAGGAGGCAGACTGGGGCTTCTTTGACGTTC	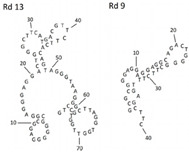	GFET	-	-	Wiedman et al. [30]

## 4. Conclusions and Perspectives

In conclusion, using SELEX, a diversity of taxa-specific aptamers has been developed for diagnosing fungal pathogens that cause invasive infections in humans. For most of these aptamers, vigorous clinical validation by independent laboratories remains to be performed. At the same time, researchers are discovering an increasing number of species -specific cell surface molecules in fungal pathogens. In addition, strain-specific biological molecules, especially those related to drug resistance, are being discovered. These newly identified molecules represent potential targets for developing novel aptamers that could be exploited for future diagnosis and/or treatment. With continued efforts in developing novel aptamers, rigorous validation of existing ones, and efforts to integrate them into various platforms, there is huge potential for developing aptamers as alternatives to traditional diagnosis and treatment of IFIs. We believe the future is bright for aptamers in the diagnosis and treatment for IFIs.

## Data Availability

The original source data reviewed by us for this manuscript are available in the cited literature.

## References

[1] Seagle E.E., Williams S.L., Chiller T.M. (2021). Recent trends in the epidemiology of fungal infections. Infect. Dis. Clin..

[2] Salehi M., Ahmadikia K., Badali H., Khodavaisy S. (2020). Opportunistic fungal infections in the epidemic area of COVID-19: A clinical and diagnostic perspective from Iran. Mycopathologia.

[3] Terrero-Salcedo D., Powers-Fletcher M.V. (2020). Updates in laboratory diagnostics for invasive fungal infections. J. Clin. Microbiol..

[4] Mendonca A., Santos H., Franco-Duarte R., Sampaio P. (2022). Fungal infections diagnosis–past, present and future. Res. Microbiol..

[5] Ahmadi A., Bashardoust B., Abdorahimi M., Aminizadeh S., Salehi M., Khodavaisy S. (2023). Emerging Challenges in Diagnosis and Treatment of Invasive Fungal Infections: Addressing the Impact of COVID-19 and New Pathogens. Curr. Fungal Infect. Rep..

[6] Yang S., Rothman R.E. (2004). PCR-based diagnostics for infectious diseases: Uses, limitations, and future applications in acute-care settings. Lancet Infect. Dis..

[7] Bouza E., Almirante B., Rodríguez J.G., Garnacho-Montero J., Salavert M., Muñoz P., Sanguinetti M. (2020). Biomarkers of fungal infection: Expert opinion on the current situation. Rev. Esp. De Quimioter..

[8] Tang X.-L., Hua Y., Guan Q., Yuan C.-H. (2016). Improved detection of deeply invasive candidiasis with DNA aptamers specific binding to (1→ 3)-β-D-glucans from Candida albicans. Eur. J. Clin. Microbiol. Infect. Dis..

[9] Byun J. (2021). Recent progress and opportunities for nucleic acid aptamers. Life.

[10] Banerjee S., Nilsen-Hamilton M. (2019). Aptamers for infectious disease diagnosis. E. Coli Infections-Importance of Early Diagnosis and Efficient Treatment.

[11] Deng B., Lin Y., Wang C., Li F., Wang Z., Zhang H., Li X.-F., Le X.C. (2014). Aptamer binding assays for proteins: The thrombin example—A review. Anal. Chim. Acta.

[12] Huang L., Tian S., Zhao W., Liu K., Ma X., Guo J. (2021). Aptamer-based lateral flow assay on-site biosensors. Biosens. Bioelectron..

[13] Davydova A., Vorobjeva M., Pyshnyi D., Altman S., Vlassov V., Venyaminova A. (2016). Aptamers against pathogenic microorganisms. Crit. Rev. Microbiol..

[14] Huang J., Chen X., Fu X., Li Z., Huang Y., Liang C. (2021). Advances in aptamer-based biomarker discovery. Front. Cell Dev. Biol..

[15] Ellington A.D., Szostak J.W. (1990). In vitro selection of RNA molecules that bind specific ligands. Nature.

[16] Krüger A., de Jesus Santos A.P., de Sá V., Ulrich H., Wrenger C. (2021). Aptamer applications in emerging viral diseases. Pharmaceuticals.

[17] Li J., Zhang Z., Liu R., Amini R., Salena B.J., Li Y. (2023). Discovery and translation of functional nucleic acids for clinically diagnosing infectious diseases: Opportunities and challenges. TrAC Trends Anal. Chem..

[18] Afrasiabi S., Pourhajibagher M., Raoofian R., Tabarzad M., Bahador A. (2020). Therapeutic applications of nucleic acid aptamers in microbial infections. J. Biomed. Sci..

[19] Hua Y., Hu F., Ren X., Xiong Y., Hu J., Su F., Tang X., Wen Y. (2023). A novel aptamer-G-quadruplex/hemin self-assembling color system: Rapid visual diagnosis of invasive fungal infections. Ann. Clin. Microbiol. Antimicrob..

[20] Clack K., Sallam M., Muyldermans S., Sambasivam P., Nguyen C.M., Nguyen N.-T. (2024). Instant Candida albicans Detection Using Ultra-Stable Aptamer Conjugated Gold Nanoparticles. Micromachines.

[21] Kneißle K., Krämer M., Kissmann A.-K., Xing H., Müller F., Amann V., Noschka R., Gottschalk K.-E., Bozdogan A., Andersson J. (2022). A Polyclonal SELEX Aptamer Library Allows Differentiation of *Candida albicans*, *C. auris* and *C. parapsilosis* Cells from Human Dermal Fibroblasts. J. Fungi.

[22] Milnes B.E. (2019). Identification and Validation of Novel Aptamers against Fungal Cells. Master’s Thesis.

[23] Seo J.-W., Kim J.Y., Oh J.-J., Kim Y.J., Kim G.-H. (2021). Selection and characterization of toxic Aspergillus spore-specific DNA aptamer using spore-SELEX. RSC Adv..

[24] Wang Y., Xu J. (2024). Associations between genomic variants and antifungal susceptibilities in the archived global *Candida auris* population. J. Fungi.

[25] Xu J. (2022). Assessing global fungal threats to humans. mLife.

[26] Fisher M.C., Alastruey-Izquierdo A., Berman J., Bicanic T., Bignell E.M., Bowyer P., Bromley M., Brüggemann R., Garber G., Cornely O.A. (2022). Tackling the emerging threat of antifungal resistance to human health. Nat. Rev. Microbiol..

[27] Santana D.J., Anku J.A., Zhao G., Zarnowski R., Johnson C.J., Hautau H., Visser N.D., Ibrahim A.S., Andes D., Nett J.E. (2023). A Candida auris–specific adhesin, Scf1, governs surface association, colonization, and virulence. Science.

[28] Bukkems L.M., van Dommelen L., Regis M., van den Heuvel E., Nieuwenhuizen L. (2023). The Use of Galactomannan Antigen Assays for the Diagnosis of Invasive Pulmonary Aspergillosis in the Hematological Patient: A Systematic Review and Meta-Analysis. J. Fungi.

[29] Bruch A., Kelani A.A., Blango M.G. (2022). RNA-based therapeutics to treat human fungal infections. Trends Microbiol..

[30] Wiedman G.R., Zhao Y., Mustaev A., Ping J., Vishnubhotla R., Johnson A.C., Perlin D.S. (2017). An aptamer-based biosensor for the azole class of antifungal drugs. mSphere.

